# A Review of COVID-19-Related Publications and Lag Times During the First Six Months of the Year 2020

**DOI:** 10.5811/westjem.2021.3.51737

**Published:** 2021-06-29

**Authors:** Christopher J. Carvalho, Matthew P. Fuller, Emmanuel A. Quaidoo, Ahson S. Haider, Jonathan J. Rodriguez, Angela H.K. Wong, Mindy M. Duong, Robert M. Rodriguez

**Affiliations:** University of California, San Francisco, Department of Emergency Medicine, San Francisco, California

## Abstract

**Introduction:**

Considering the need for information regarding approaches to prevention and treatment of coronavirus disease 2019 (COVID-19), we sought to determine publication lag times of COVID-19-related original research articles published in top general medicine and emergency medicine (EM) journals. We further sought to characterize the types of COVID-19 publications within these journals.

**Methods:**

We reviewed 125 top-ranked general medicine journals and 20 top-ranked EM-specific journals for COVID-19-related publications. We abstracted article titles and manuscript details for each COVID-19-related article published between January 1–June 30, 2020, and categorized articles as one of the following: original research; case report; review; or commentary. We abstracted data for preprint publications over the same time period and determined whether articles from the general medicine and EM journals had been previously published as preprint articles. Our primary outcomes were the following: 1) lag time (days) between global cumulative World Health Organization (WHO)-confirmed cases of COVID-19 and publications; 2) lag times between preprint article publication and peer-reviewed journal publication; and 3) lag times between submission and publication in peer-reviewed journals. Our secondary outcome was to characterize COVID-19-related publications.

**Results:**

The first original research publications appeared in a general medicine journal 20 days and in an EM journal 58 days after the first WHO-confirmed case of COVID-19. We found median and mean lag times between preprint publications and journal publications of 32 days (19, 49) and 36 days (22) for general medicine journals, and 26 days (16, 36) and 25 days (13) for EM journals. Median and mean lag times between submission and publication were 30 days (19, 45) and 35 days (13) for general medicine journals, and 23 days (11, 39) and 27 days (19) for EM journals. Of 2530 general medicine journal articles and 351 EM journal articles, 28% and 23.6% were original research. We noted substantial closing of the preprint to peer-reviewed publication (160 days pre-pandemic) and peer-reviewed journal submission to publication (194 days pre-pandemic) lag times for COVID-19 manuscripts.

**Conclusion:**

We found a rapid and robust response with shortened publication lag times to meet the need for the publication of original research and other vital medical information related to COVID-19 during the first six months of 2020.

## INTRODUCTION

The first reports of coronavirus disease 2019 (COVID-19) surfaced in December 2019. COVID-19 has infected nearly 173 million people and claimed more than 3.7 million lives globally as of June 7, 2021, ushering in a need for rapid dissemination of information and original research regarding approaches to prevention and treatment.^1^,^2^ Given this need for critical information, we sought to determine publication lag times of COVID-19-related original research articles published in the top-ranked 125 general medicine journals and the top 20 emergency medicine (EM) journals during the first six months of the year 2020. We further sought to characterize the types of COVID-19-related publications in these top-ranked journals.

## METHODS

We abstracted data regarding World Health Organization (WHO)-confirmed COVID-19 cases and deaths from the WHO COVID-19 dashboard.^1^ We reviewed the 125 top-ranked, peer-reviewed journals under the category of “medicine,” as ranked by the *Scimago* Journal & Country Rank website for articles published between January 1–June 30, 2020.^3^ We included all journals listed in this category, regardless of focus (clinical vs lab) and excluded the one journal that required login to access article titles and abstracts. No EM journals were ranked in the top 125 general medicine category. We reviewed the 20 top-ranked, peer-reviewed EM journals as ranked in a recent EM journal review for the same time period.^4^

### Outcomes

Focusing on original research articles from the top peer-reviewed general medicine and EM journals, our primary outcome was to determine lag times (days) between 1) global cumulative WHO-confirmed cases of COVID-19 and peer-reviewed journal publications; 2) preprint publication of articles and publication in peer-reviewed journals; and 3) submissions to peer-reviewed journals and publication within these journals. Our secondary outcome was to characterize COVID-19-related publications.

We performed data collection using a systematic approach designed by the senior and lead author, who generated a written template and algorithm for data abstraction. They conducted individual and group orientation meetings with the other authors. The lead author conducted weekly meetings to assure continued consistency, and reviewed samples of the data with the other abstractors for real-time data quality assurance. We searched all the remaining 144 journals’ official publication websites using their embedded search functions. We used the keywords “COVID-19” OR “SARS-CoV-2” OR “coronavirus” to abstract article titles and manuscript details, including date of publication, date of submission, and primary author’s country affiliation. When available on some websites, a “COVID-19 collection” of articles was used for article abstraction in leu of a keyword search. We included all articles in the keyword and “COVID-19 collections” searches.

We screened and abstracted articles according to standard definitions of study designs derived from the *JAMA* “Instructions for Authors: Determine my Study Type”: original research (presentation of original data); case report (single patient presentation); review (literature summary on a given topic); or commentary (correspondence, editorials, perspectives, news, and proposed guidelines). We further classified original research articles into the following categories (more than one option applicable): case series; case-control; cohort (retrospective and prospective); cross-sectional survey; randomized control trial (RCT); drug trial; basic science/laboratory; epidemiological; or observational-other. We defined epidemiological studies as those that focused on surveillance, modeling or tracking the spread of COVID-19. We defined “observational-other” as prospective and retrospective observational study designs not meeting the standard, aforementioned observational design definitions. Most of these studies were published as correspondence (letters). Categorizations were reviewed by the lead and senior author.

We abstracted data for preprint publications from the Dimensions database (Digital Science & Research Solutions Ltd, London, England), a repository using artificial intelligence and machine learning to compile information pertaining to the complete research cycle, over the same time period (January 1–June 30, 2020).^5^ We screened the Dimensions data abstracted and determined whether articles from the top general medicine and EM journals had been previously published as preprint articles.

We calculated median (interquartile range [IQR]) and mean (standard deviation [SD]) lag times between article preprint publication and peer-reviewed journal publication, and between article submission and publication in peer-reviewed journals. We did not intend to compare lag times between the two groups of journals (general medicine and EM), and therefore did not perform hypothesis testing or other statistical comparisons.

Because of the exponential increase in numbers over time, we present data regarding total numbers of COVID-19 cases, deaths, and publications on a logarithmic scale to allow for more practical visual inspection. We included COVID-19 deaths in the figure to add another critical perspective regarding the burden of the pandemic, although not explicitly measured in our original outcomes.

## RESULTS

### 125 Top-Ranked General Medicine Journals

The first three original research articles were published on January 24, 2020, 20 days after the first WHO-confirmed case of COVID-19.^1^ The median (IQR) and mean (SD) lag times between preprint publication and peer-reviewed journal publication were 32 days (19, 49) and 36 days (22). The median and mean lag times between submission to and publication in peer-reviewed journals were 30 days (19, 45) and 35 days (13). Data for lag times was normally distributed.

Of the 2,530 COVID-19-related articles published from January 1–June 30, 2020 in the 125 top general medicine journals, 1565 (61.9%) were commentaries, 709 (28.0%) were original research, 173 (6.8%) were reviews, and 83 (3.3%) were case reports. We found 74 unique countries of primary author affiliation, most commonly the United States (40%), the United Kingdom (16.7%), and China (13.4%) (Table 1). Of the 709 original research articles, the top three study designs were observational-other (205 [28.9%]), epidemiological (124 [17.5%]), and case-series (103 [14.5%]). Of the 17 clinical trials published, 10 (1.4% of all original research articles) were randomized control trials (RCT); the other seven clinical trials were non-randomized drug trials. One hundred and eight (15.2%) of the original research publications were previously published on preprint servers, and 282 (37.8%) had article original submission dates publicly available.

### 20 Top-Ranked Emergency Medicine Journals

The first original research article in an EM journal was published on March 2, 2020, 58 days after the first WHO-confirmed case of COVID-19.^1^ The median (IQR) and mean (SD) lag times between preprint publication and EM journal publication were 26 days (16, 36) and 25 days (13). The median and mean lag times between article submission and publication within EM journals were 23 days (11, 39) and 27 days (19). Data for lag times was normally distributed.

Of the 351 COVID-19-related articles published from January 1–June 30, 2020 in the 20 top EM journals, 191 (54.4%) were commentaries, 83 (23.6%) were original research, 49 (14%) were reviews, and 28 (8%) were case reports. We found 28 unique countries of primary author affiliation, most commonly the United States (40.7%), Italy (13.7%), and Canada (10.3%) (Table 2). Of the 83 original research articles, the top three study designs were observational-other (41 [49.4%]), cohort (13 [15.7%]), and survey (8 [9.6%]). We found only one (1.2%) clinical trial (non-randomized). Nine (10.8%) of the original research publications were previously published on preprint servers, and 67 (80.7%) had article original submission dates publicly available.

In the figure we present a graph of numbers of cumulative global COVID-19 cases, deaths, top 125 general medicine journal articles, top 20 EM journal articles, original research general medicine and EM journal articles, and preprint articles plotted logarithmically across the first six months of the year 2020. The figure demonstrates relatively symmetric and parallel curves with the slopes of the top 125 general medicine and EM journal original research publications lagging roughly one and three months behind the pandemic curve, respectively.

## DISCUSSION

In this review of COVID-19-related publications in top general medicine and EM journals we found a rapid and robust response to meet the need for original research and other vital medical information during the first six months of 2020. Within one month, COVID-19 publications in the top 125 general medicine journals skyrocketed and the slopes of subsequent publications mirrored the slope of the pandemic. Emergency medicine journal publications and EM journal original research publications lagged roughly two and three months behind the pandemic curve, respectively. While original research constituted just one quarter of all COVID-19 journal article publications, the rapidity of its production remains nevertheless impressive.

Given their greater complexity, the lack of early RCTs is not surprising. The first randomized controlled drug trial, which evaluated the effectiveness of a lopinavir–ritonavir combination, was published on March 18, 2020, 74 days after the first WHO-confirmed case. Two more RCTs were published in April, five in May, and two in June. The first and only clinical trial within the EM-specific journals (evaluating the effectiveness of plasma taken from convalescent donors) was published on May 28, 2020, 145 days after the first WHO-confirmed case.

A number of mechanisms are available to accelerate dissemination of critical research findings. Prior to or in tandem with submission to peer-reviewed journals, investigators can choose to publish their work on preprint servers for immediate dissemination. Concerns about inadequate review and controls for validity notwithstanding, preprint publication may afford investigators the added benefits of gaining feedback and claiming provenance of an idea.^6^ Journal editors can also accelerate publication and rejection of manuscripts by leveraging *fast-tracking* protocols.^7^ Although we did not find specific language in journal mastheads regarding fast-tracking of COVID-19 articles, journal editors and reviewers may have informally adopted this practice.

Investigating the lag time between preprint publication and journal publication dates, Herbert et al evaluated 8711 articles published on the preprint repository bioRxiv in 2019 and found a median lag time of 160 days.^8^ We found only 15.2% and 10.8% of original research articles published in general medicine and EM journals had been deposited on preprint repositories. However, a closing of the preprint-peer review publication gap for COVID-19 manuscripts was noted with significantly shorter median lag times of 32 days and 26 days for general medicine and EM journals, respectively, consistent with the findings of Krumholz et al (46 days for COVID-19 vs 141 days for non-COVID-19 papers).^9^

There is little prior literature regarding baseline lag times before the COVID-19 pandemic. In 2019, Shan et al found a median lag time between article submission and publication of 194 days for articles published in the *British Medical Journal* (included in the top 125 general medicine journals).^10^ In terms of EM journals, the only relevant study is one we published in which we characterized a different metric – median decision times (time from submission to a decision).^4^ Nevertheless, the relatively short 30-day general medicine journal and 23-day EM journal median lag times between initial submission and publication date suggest that journals are expediting reviews and publication decisions, either through a formal fast-track process or otherwise.

The longer delay for original research to appear in EM-specific journals may be due to investigators’ customary submission process of starting with the highest impact factor journals and working their way down – most of the top 125 journals have substantially higher impact factors than the EM journals.^4^ Drawing articles from all fields of medicine and public health instead of just EM-related topics, the broader scope of general medicine journals may also contribute to faster emergence of COVID-19 publications in these journals.

## LIMITATIONS

The main limitation of this work is that we only reviewed articles from the top journals as assessed by one organization (*Scimago*) and one group of EM investigators. Sixty-two general medicine and six EM journals published fewer than five COVID-19-related articles. Numbers of COVID-19 articles, percentages of original research, and lag times may differ for publications in other medical journals not on these lists. Another limitation, as mentioned above, is that we do not have standardized true baselines for pre-COVID-19 pandemic lag times. Additionally, although the lead and senior authors reviewed the other abstractors’ categorizations, these classifications may still be subjective and we did not calculate inter-rater reliability. Finally, the over-representation of observational study designs in COVID-19 publications may skew the data toward shorter median lag times.

## CONCLUSION

We found remarkably short publication lag times at the early stage of the COVID-19 pandemic, indicating that journal editors and reviewers responded appropriately to the need for vital information. Yet with over 13,000 worldwide COVID-19-related deaths per day on average in January 2021,^1^ editors and journal managers should seek to streamline review and publishing processes even further. If the speed of the peer-review process has reached its ceiling, preprint publications may serve to bridge the critical need for relevant information during times of medical crisis.

## Figures and Tables

**Figure:**
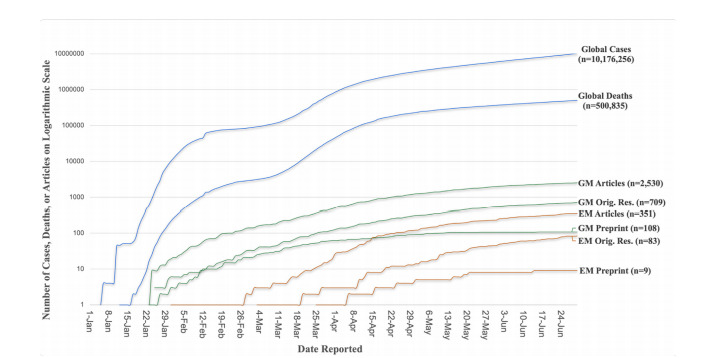
Global COVID-19 cases and deaths compared to journal publications over time. GM articles, 125 top general medicine journal article publications; GM Orig. Res., 125 top general medicine journal original research article publications; GM Preprint, 125 top general medicine journal original research articles published as preprint articles; EM Articles, 20 top emergency medicine (EM) journal articles published; EM Orig. Res., 20 top EM journal original research articles published; EM Preprint, 20 top EM journal original research articles published as preprint articles.

**Table 1:** Characteristics of the 125 top-ranked general medicine journal articles published between January 1, 2020 and June 30, 2020.

	n (%)
General Medicine Journal Article Type (n=2,530)	
Commentary	1,565 (61.9)
Original research	709 (28.0)
Observational-other	205 (28.9)
Epidemiological	124 (17.5)
Case Series	103 (14.5)
Cohort	80 (11.3)
Basic Science/laboratory	79 (11.1)
Survey	56 (7.9)
Case control	25 (3.5)
Cross sectional	20 (2.8)
Clinical trial	17 (2.4)
Review	173 (6.8)
Case report	83 (3.3)
Primary author country of origin	Articles published
United States	1,011 (40.0)
United Kingdom	422 (16.7)
China	339 (13.4)
Italy	190 (7.5)
France	74 (2.9)
Canada	69 (2.7)
Germany	56 (2.2)
Singapore	45 (1.8)
Switzerland	38 (1.5)
Spain	34 (1.3)

**Table 2:** Characteristics of the 20 top-ranked emergency medicine journal articles published between January 1, 2020 – June 30, 2020.

	n (%)
Emergency Medicine Journal Article Type (n=351)	
Commentary	191 (54.4)
Original research	83 (23.6)
Observational-other	41 (49.4)
Cohort	13 (15.7)
Survey	8 (9.6)
Basic Science/laboratory	7 (8.4)
Case Series	6 (7.2)
Cross sectional	4 (4.8)
Case control	2 (2.4)
Epidemiological	1 (1.2)
Clinical trial	1 (1.2)
Review	49 (14.0)
Case report	28 (8.0)
Primary author country of origin	Articles published
United States	143 (40.7)
Italy	48 (13.7)
Canada	36 (10.3)
China	18 (5.1)
Spain	13 (3.7)
United Kingdom	11 (3.1)
France	10 (2.8)
Taiwan	9 (2.6)
Australia	8 (2.3)
India	8 (2.3)
